# Diabetes alone should not be a reason for withholding adjuvant chemotherapy for stage III colon cancer

**DOI:** 10.15256/joc.2011.1.7

**Published:** 2011-12-27

**Authors:** Maren A. van Waalwijk, Saskia A. M. van de Schans, Harm R. Haak, Martine Extermann, Wouter M. W. Dercksen, Maryska L. G. Janssen-Heijnen

**Affiliations:** ^1^Department of Internal Medicine, Máxima Medical Centre, Eindhoven, The Netherlands; ^2^Department of Research, Eindhoven Cancer Registry, Comprehensive Cancer Centre South, Eindhoven, The Netherlands; ^3^Moffitt Cancer Center, Department of Senior Adult Oncology, University of South Florida, Tampa, FL, USA; ^4^Department of Clinical Epidemiology, VieCuri Medical Centre Venlo, The Netherlands

**Keywords:** colon cancer, comorbidity, diabetes mellitus, population-based, survival, treatment

## Abstract

**Background:**

With increasing prevalence of diabetes mellitus and colon cancer, the number of patients suffering from both diseases is growing, and physicians are being faced with complicated treatment decisions.

**Objective:**

To investigate the association between diabetes and treatment/course of stage III colon cancer and the association between colon cancer and course of diabetes.

**Materials and Methods:**

Additional information was collected from the medical records of all patients with both stage III colon cancer and diabetes (*n*=201) and a random sample of stage III colon cancer patients without diabetes (*n*=206) in the area of the population-based Eindhoven Cancer Registry (1998–2007).

**Results:**

Colon cancer patients without diabetes were more likely to receive adjuvant chemotherapy compared with diabetic colon cancer patients (OR 1.8; 95% CI 1.2–2.7). After adjustment for age, this difference was borderline significant (OR 1.6; 95% CI 1.0–2.6). Diabetic patients did not have: significantly more side-effects from surgery or adjuvant chemotherapy; more recurrence from colon cancer; significantly shorter time interval until recurrence; or a poorer disease-free survival or overall survival. Age and withholding of adjuvant chemotherapy were most predictive of all-cause mortality. After colon cancer diagnosis, the dose of antiglycaemic medications was increased in 22% of diabetic patients, resulting in significantly lower glycaemic indexes than before colon cancer diagnosis.

**Conclusions:**

Since diabetic patients did not have more side-effects of adjuvant chemotherapy, and adjuvant chemotherapy had a positive effect on survival for both patients with and without diabetes, diabetes alone should not be a reason for withholding adjuvant chemotherapy.

Journal of Comorbidity 2011;1:19–27

## Introduction

Due to ageing of the population and the increasing prevalence of obesity, the incidence of diabetes mellitus is rising [1, 2], and, at the same time, an increase in the incidence of colon cancer is also being observed. In the Netherlands, for example, the incidence rate [European standardized rate (ESR)] of colon cancer increased from 29 in 1989 to 36 per 100,000 person-years in 2006 [3]. Therefore, the number of patients with both colon cancer and diabetes is increasing. Previous studies have shown a worse prognosis for colon cancer patients with diabetes compared with those without diabetes [4–7]. Several factors might be responsible for this effect: the increased risk of death due to diabetes or accompanying comorbid conditions; neglect of treatment of diabetes; more contraindications for anticancer treatment; lower effectiveness of adjuvant chemotherapy in diabetic patients; or a higher rate of treatment-related complications.

The current standard treatment for stage III colon cancer is surgery with adjuvant chemotherapy. However, as shown in a previous study, colon cancer patients with diabetes received adjuvant chemotherapy less frequently compared with those without diabetes [8]. This implies that colon cancer patients with diabetes might have a poorer prognosis due to them not receiving appropriate treatment for their colon cancer. On the other hand, diabetic patients might be more prone to chemotherapy-related toxicity because of the high prevalence of pre-existing comorbid conditions in this group. Only a few studies have analysed the effect of diabetes on colon cancer treatment and treatment outcome [5, 6, 8]. Even less is known about the effect of colon cancer on the course and outcome of diabetes.

We studied the association of diabetes with the administration of adjuvant chemotherapy, dose, treatment-related toxicity, and outcome of treatment in stage III colon cancer patients. In addition, the association between colon cancer and its treatment, on the one hand, and the course of diabetes, on the other, are described.

## Materials and Methods

### Study population and data collection

Data from patients with stage III colon cancer diagnosed in the registration area of the Eindhoven Cancer Registry were used. This registry records data on all patients with newly diagnosed cancer in the southern part of the Netherlands (an area with 2.4 million inhabitants, 10 general hospitals, and two radiotherapy institutes). Registration clerks actively collect data on diagnosis, topography, histology, stage, and information about initial treatment (delivered within 6 months from diagnosis) from hospital medical records. Since 1993, the presence of comorbidity at the time of diagnosis has also been documented. Specific comorbid conditions were registered as dichotomous variables (Yes/No), according to the medical history of the patient, use of relevant drugs, and diagnostic work-up. Conditions included chronic obstructive pulmonary disease (COPD), cardiovascular disease (CVD), peripheral arterial disease, cerebrovascular disease, other malignancies, and hypertension, as described in a previous publication [9]. In our study, the specific comorbid condition (CVD) and the total amount of comorbidities (minus diabetes) were used to study the different patient characterizations between colon cancer patients with and without diabetes (Table 1). Furthermore, the number of comorbidities (minus diabetes) was analysed in a multivariable survival analysis. The specific comorbidities of CVD and COPD were evaluated in the subgroup analysis of patients who died before adjuvant chemotherapy could be administered.

CVD included: any previous myocardial infarction; current treatment for cardiac insufficiency, angina pectoris, and peripheral arterial disease; previous coronary artery bypass graft surgery; and current or previous treatment for cerebrovascular disease.

We selected all the colon cancer patients with stage III disease, who were diagnosed between 1998 and 2007, aged 50 years and older, and who had undergone surgical treatment. For this study, we included all patients with both stage III colon cancer and diabetes (*n*=236) and a random sample of stage III colon cancer patients without diabetes (*n*=240).

Additional information on patient characteristics, treatment, and outcome was collected from the medical records. The medical record is regarded as the most complete source of information on a patient’s past and current health status [10]. Patient characteristics included information on overall performance according to the Eastern Cooperative Oncology Group (ECOG) performance scale [11], body mass index (BMI), diabetes status, and smoking status. ECOG score was registered in two ways. If present, we used the World Health Organization (WHO) performance scale, ECOG score or a specific remark describing performance status recorded by the specialist. If not present, we estimated the ECOG score from the text in the medical record. BMI was classified as underweight (<18 kg/m^2^), normal (18–25 kg/m^2^), overweight (26–30 kg/m^2^), and obese (>30 kg/m^2^).

Information about diabetes status was collected, including type of diabetes, year of onset, and the existence of diabetic complications. Type of diabetes was directly registered from the medical record. When data were inconsistent or conflicting (e.g. patients registered as having type 2 diabetes with a low BMI or diagnosed at a young age) diabetes status was recorded as unknown. We registered microvascular complications (retinopathy, nephropathy, and neuropathy) and macrovascular complications (coronary diseases and peripheral arterial disease) at the time of colon cancer diagnosis. Macrovascular complications were recorded as diabetes-related if they occurred after the onset of diabetes. Duration of diabetes was defined as from year of onset of diabetes to diagnosis of colon cancer. To study the effect of colon cancer on the course of diabetes, haemoglobin A1c (HbA1c) regulation and the use of medication were registered before and after treatment of colon cancer. In the Netherlands, most diabetic patients are treated by their general practitioner (GP). We therefore contacted the patients’ GPs if any of this information was missing or incomplete in the medical record.

Detailed information on treatment of colon cancer was collected, including adjuvant chemotherapy, type of adjuvant chemotherapy, reason for withholding adjuvant chemotherapy, number and length of cycles, possible dose adjustments, and reason for dose adjustments. In addition, treatment complications (from surgery or adjuvant chemotherapy) were registered. Toxicity was recorded using the Common Toxicity Criteria [11]. Only serious side-effects (Grade 3 or 4 toxicity) were registered.

To study the effect of diabetes on the prognosis of colon cancer, we collected information on recurrence rate and vital status. Recurrence was defined as the existence of local recurrence, metastatic disease, or both, as noted in the medical record. Time to recurrence was defined as the time from colon cancer diagnosis to recurrence.

Information about vital status was actively obtained from the municipal personal records database on January 2010. Overall survival (OS) was defined as the time from diagnosis to death or the end of follow-up. Disease-free survival was defined as the time from diagnosis to recurrence, death, or end of follow-up, whichever came first. Patients who were still alive at the end of follow-up were censored on the date of last follow-up.

A total of 476 patients who met our inclusion criteria were selected from the Eindhoven Cancer Registry database. The medical records were missing or did not contain sufficient data for 69 of these patients (35 patients with diabetes, 34 patients without diabetes), so they were excluded from the study. The excluded patients did not differ in age, number of comorbid conditions, or number of deaths compared with patients who were included. This brought the total number of patients included in our study to 407. We handled the data from our cancer registry according to the officially recognized code of conduct ‘Use of data in health research’ [12]. Patient consent was not required as general information from the cancer registry was at the hospital level and the data were made anonymous.

### Statistical analyses

First, we determined whether characteristics differed significantly between diabetic and non-diabetic patients. Second, colon cancer patients with and without diabetes were compared with respect to the proportion of patients receiving adjuvant chemotherapy, regimen, dose reduction, and reason for dose reduction. Furthermore, we compared both groups with respect to treatment response, postoperative complications (as a proportion of all patients who underwent surgery), and toxicity from adjuvant chemotherapy (as a proportion of all patients who received adjuvant chemotherapy). To analyse the aforementioned associations, we used percentages and the Chi-square test. Because diabetic patients differed significantly in age, comorbidity, and BMI, we adjusted for these variables in multivariable logistic regression analyses.

Hazard ratios (HR) for all-cause mortality were estimated for diabetic patients, with non-diabetic patients as a reference. To evaluate whether a possible difference in survival between diabetic and non-diabetic patients was confounded by differences in patient characteristics or treatment, we used a Cox regression analysis including age, ECOG score, gender, adjuvant chemotherapy administration, and comorbidity. We also tested for interaction with age or adjuvant chemotherapy. To evaluate the influence of the severity of diabetes, we analysed survival for different HbA1c groups and duration of diabetes. We also evaluated the prognostic effect of using metformin or insulin for diabetes.

In addition, the association between colon cancer and treatment/course of diabetes was analysed by evaluating changes in the treatment of diabetes and glycaemic control before and after colon cancer diagnosis. For the mean HbA1c before and after colon cancer diagnosis, we used the paired *t*-test. The SAS computer package (version 9.1) was used for all statistical analyses (SAS Institute Inc., Cary, North Carolina, USA, 1999).

## Results

Table 1 shows the baseline characteristics of patients according to diabetes status. Patients with diabetes were slightly older than patients without diabetes, but this was not statistically significant (*p*=0.12). BMI was significantly higher in diabetic patients (*p*=0.02). Diabetic patients had a significantly poorer performance status (*p*<0.01) and significantly more comorbid conditions (*p*<0.01), especially CVD (*p*<0.01).

### Adjuvant chemotherapy

In all, 208 patients (51%) received adjuvant chemotherapy. Fifty-nine percent of the adjuvant chemotherapeutic regimens included 5-fluorouracil (5-FU) and leucovorin. Other regimens were capecitabine as monotherapy or in combination with oxaliplatin (20%), or 5-FU, leucovorin, and oxaliplatin according to the FOLFOX scheme (12%). The distribution of regimens did not differ for patients with or without diabetes. Sixty percent of patients without diabetes versus 42% of those with diabetes received adjuvant chemotherapy [odds ratio (OR) 1.8; 95% confidence interval (CI) 1.2–27]. After adjustment for age, this difference was only borderline significant (OR 1.6; 95% CI 1.0–2.6). Age was not an effect modifier. Therefore, the effect of diabetes on receiving adjuvant chemotherapy was not statistically different in the two age groups. Median age was 67 years for patients receiving adjuvant chemotherapy versus 77 years for patients not receiving adjuvant chemotherapy. This was similar for patients with and without diabetes.

### Reasons given for withholding adjuvant chemotherapy

Advanced age was the most common reason given for withholding adjuvant chemotherapy (22% for patients with diabetes and 29% for those without diabetes) (Table 2). Patient’s choice was the second most common reason (17 and 15% for those with and without diabetes, respectively). Comorbidity was more often a reason given for withholding adjuvant chemotherapy in diabetic patients compared with non-diabetic patients (15 vs. 2%, respectively). Twenty-one patients had already died before adjuvant chemotherapy could be commenced. This was the most likely reason why these patients did not receive adjuvant chemotherapy (11% in diabetic patients and 10% in non-diabetic patients). For patients who had died before adjuvant chemotherapy could be commenced, more had diabetes [13 vs. 8 patients (62 vs. 38%), respectively], pulmonary disease [4 vs. 45 patients (19 vs. 12%), respectively] and CVD [10 vs. 139 patients (48 vs. 36%), respectively], compared with those who had started adjuvant chemotherapy.

### Adaptations to adjuvant chemotherapy

The dose of adjuvant chemotherapy was reduced in 89 of 208 patients (43%) receiving adjuvant chemotherapy. This was similar for diabetic and non-diabetic patients. The most important reasons given for dose reductions were gastrointestinal side-effects [29 patients (33%)] and neurological side-effects [18 patients (20%)]. There were no differences between diabetic and non-diabetic patients with respect to reasons given for dose reductions.

### Complications

Diabetic patients tended to have more complications than non-diabetic patients from surgery [62 vs. 48 patients (31 vs. 23%), respectively; *p*=0.09]. There were no significant differences in total toxicity from adjuvant chemotherapy [31 diabetic patients (15%) versus 41 non-diabetic patients (20%); *p*=0.19] or type of toxicity (neuropathy or infections). However, more diabetic than non-diabetic patients were admitted to the intensive care unit because of surgical or adjuvant chemotherapy-related complications [24 diabetic patients (12%) vs. 8 non-diabetic patients (4%); *p*<0.01].

### Association between diabetes and recurrence of colon cancer

In 164 patients (42%), a recurrence of colon cancer occurred (Table 3). This occurred in 75 diabetic patients (39%) and in 89 non-diabetic patients (45%) (*p*=0.26). Seventy-nine of the patients who received adjuvant chemotherapy (39%) had recurrent disease. Again, there was no significant difference between patients with or without diabetes. There was no statistically significant difference in median time to recurrence in diabetic versus non-diabetic patients (19 vs. 22 months, respectively; *p*=0.45). There was also no significant difference in median time to recurrence between patients who received adjuvant chemotherapy and those who did not (19 vs. 21 months, respectively; *p*=0.41).

### Association between diabetes and survival of colon cancer patients

**Figure 1:**
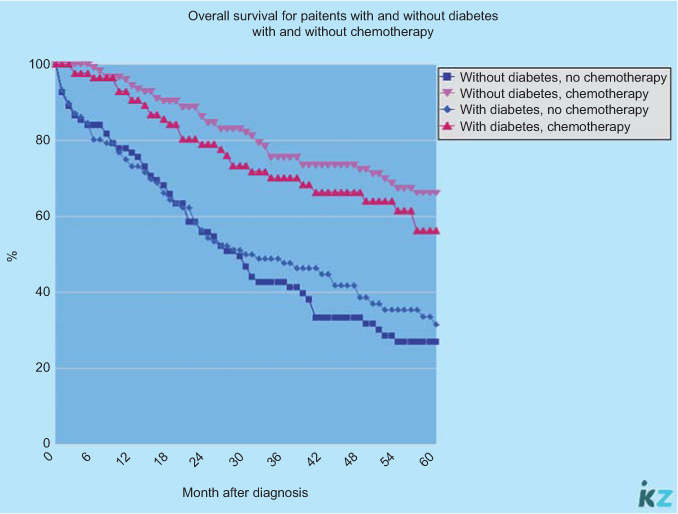
Overall survival for colon cancer patients with and without diabetes, according to chemotherapy.

At the end of follow-up, 201 patients (49%) were still alive, 205 patients (50%) had died and one was lost to follow-up. Five-year OS was slightly better for non-diabetic patients (50%) compared with diabetic patients (42%) (borderline significant, *p*=0.05) (Figure 1). Table 4 shows univariate and multivariable survival analyses. In the univariate analysis, the HR for all-cause mortality for diabetic patients versus non-diabetic patients was 1.3 (borderline significant: 95% CI 0.99–1.72). However, after adjustment for other prognostic parameters, this effect disappeared. There was no interaction with age or adjuvant chemotherapy. CVD alone did not influence survival. Patients who received adjuvant chemotherapy were significantly less likely to die than those who did not receive adjuvant chemotherapy, even after adjustment for age, diabetes status, ECOG score, gender, and number of comorbid conditions (HR 0.52). We also calculated HR for all-cause mortality for different parameters of diabetes. Mortality was not significantly associated with glycaemic control, duration of diabetes, use of metformin, or use of insulin (Table 5).

**Figure 2:**
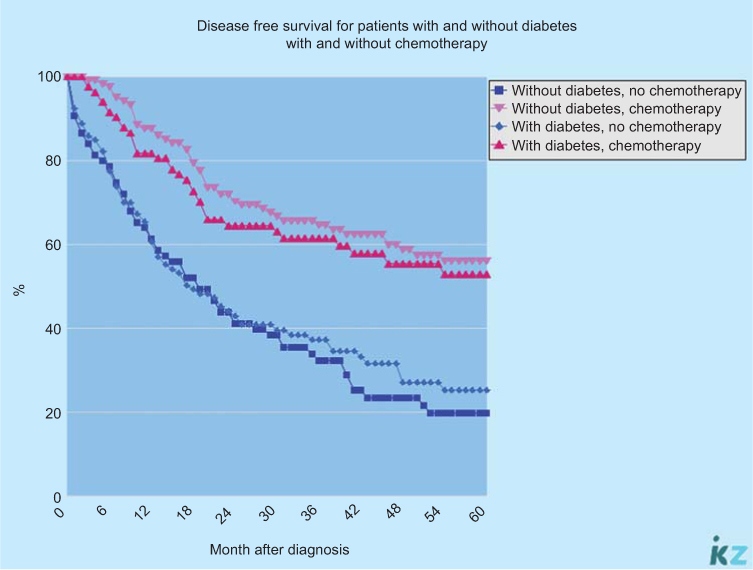
Disease-free survival for colon cancer patients with and without diabetes, according to chemotherapy.

Disease-free survival was calculated for diabetic patients versus non-diabetic patients (Figure 2). There was no significant difference between these groups (*p*=0.1), HR 1.24 (95% CI: 0.95–1.6) in the multivariate analysis. Disease-free survival was significantly longer for patients receiving adjuvant chemotherapy (*p*<0.01), HR 0.40 (95% CI: 0.30–0.52) in the multivariate analysis.

### Influence of colon cancer on diabetes

At the time of colon cancer diagnosis, 192 patients had type 2 diabetes, and one patient had confirmed type 1 diabetes. Diabetes status was unknown for eight patients. Another 11 patients (5%) developed diabetes after colon cancer diagnosis. Median duration from diabetes to colon cancer diagnosis was 5.5 years with a minimum duration of 2 months and a maximum duration of 50 years. Information on glycaemic control was available for 90 patients. Mean HbA1c 1 year before diagnosis of colon cancer was 7.6% and 1 year after diagnosis was 7.2% (*p*<0.01). Mean decrease in HbA1c was 0.38%. Before colon cancer diagnosis, 135 patients (67%) were treated with an oral antiglycaemic agent (Table 6). Seventy-six patients (50%) were treated with metformin as monotherapy or in combination therapy with another oral antiglycaemic agent. One year after colon cancer diagnosis, one patient who formerly used an oral antiglycaemic agent, switched to insulin. Twenty-two patients (16%) who used oral antiglycaemic agents received a higher dose and 14 patients (33%) who used insulin received a higher dose after colon cancer diagnosis.

Of all 201 diabetic patients, 80 (40%) had one or more diabetes-related complications at the time of colon cancer diagnosis. In this group, 27 patients (34%) had only microvascular complications and 20 patients (25%) had microvascular and macrovascular complications. After colon cancer diagnosis and treatment, eight new patients had developed microvascular and/or macrovascular complications.

## Discussion

This population-based study revealed that stage III colon cancer patients with diabetes had slightly more postoperative complications and were slightly less likely to receive adjuvant chemotherapy than their non-diabetic counterparts. Diabetes had no independent influence on recurrence or survival. As suspected, patients both with or without diabetes who received adjuvant chemotherapy survived longer, even after adjustment for patient characteristics. One year after surgery for colon cancer, diabetic patients had lower HbA1c than before colon cancer diagnosis.

Only a few other studies have investigated the association between diabetes mellitus and chemotherapy. Gross and colleagues also found that diabetic patients were less likely to receive adjuvant chemotherapy for stage III colon cancer, even after adjustment for patient characteristics and cancer characteristics [8]. In accordance with other studies, we found that high age was the most common reason given for withholding adjuvant chemotherapy, whereas comorbidity was reported less often [6, 13], and that the patient’s preference was also a common reason for withholding adjuvant chemotherapy (no difference between diabetic and non-diabetic patients) [14].

Some studies have investigated the recurrence rate and OS for colon cancer in diabetic patients, but the results are conflicting [5, 7, 15]. In contrast to our study, Meyerhardt and colleagues reported poorer recurrence-free survival and higher colon cancer recurrence rates for diabetic patients than for non-diabetic patients. A possible explanation for this difference might be the longer follow-up in their study (mean 4 years in our study compared with 9.4 years in their study). Moreover, in the study by Meyerhardt and colleagues, the recurrence from colon cancer was especially high for diabetic patients after 4 years. In contrast to other studies, we did not find a statistically significant effect of diabetes on OS [5, 7, 15]. These studies, however, also had larger study populations and longer durations of follow-up. In addition, some of these studies included patients with both stage II and III colon cancer, whereas we only included stage III. The effect of diabetes on outcome of colon cancer was shown to be most evident for diabetic patients with stage II colon carcinoma [5, 15]. A possible stage-dependent effect needs further research. Another likely explanation for these conflicting results is that OS for diabetic patients is partly affected by comorbidities. In our study, diabetic patients had significantly more comorbidities than non-diabetic patients, and previous studies have also shown that comorbidities negatively influence colon cancer prognosis [9, 16]. Moreover, previous studies that reported a worse OS for diabetic patients did not correct for comorbidities [5, 15].

In contrast to other studies that reported on survival of colon cancer in diabetic patients, we also studied the effect of diabetes-related prognostic variables on survival from colon cancer. The extent of diabetes in terms of duration or glycaemic control is possibly an important factor for colon cancer survival. Hyperinsulinaemia is prospectively related to colon cancer risk, distant recurrence, and death in cancer patients and higher mortality in diabetic patients [17–20]. This could imply that diabetic patients with high insulin might have a worse cancer-specific prognosis. We did not find a statistically significant effect of these variables on OS. However, data for these variables were incomplete and further research on these effects is necessary. According to *in vitro* and *in vivo* studies, metformin could have a positive effect on OS in cancer patients with diabetes and colorectal cancer, in particular [21–23]. We also found lower all-cause mortality in patients who used metformin, but this result was not significant, possibly due to the small study population (HR 0.73; 95% CI 0.49–1.11). However, this could have influenced OS in colon cancer patients with diabetes compared with non-diabetic patients.

Whether diabetes independently influences chemotherapy-related complications is doubtful. In our study, diabetic patients did not have more side-effects from adjuvant chemotherapy compared with non-diabetic patients. This is in line with the findings of an observational study of Gross and colleagues who reported that diabetic patients were not more frequently hospitalized for chemotherapy-related complications. In contrast, a previous randomized trial of colon cancer patients who received adjuvant chemotherapy found significantly more treatment-related diarrhoea in diabetic patients [5]. Although we did not find a higher risk of toxicity from chemotherapy in diabetic patients, we did find a higher rate of postoperative complications and more frequent admissions to the intensive care unit in diabetic colon cancer patients. Diabetic patients may be more prone to these complications because of bad wound healing and a weaker immune system [24–26]. Moreover, this could imply that complications are more severe in diabetic patients.

This is the first study that has investigated the association between diagnosis/treatment of colon cancer and the course of diabetes. HbA1c was significantly lower after diagnosis of colon cancer than before diagnosis of colon cancer (mean HbA1c 7.2 vs. 7.6%). This might suggest a favourable effect of colon cancer and its treatment on glycaemic control. A possible explanation is a better follow-up of patients after colon cancer since a substantial proportion of patients had higher dose medication for diabetes after colon cancer diagnosis. In addition, weight loss because of colon cancer and its treatment could lead to lower HbA1c levels.

We should keep in mind that this is a retrospective observational study in which there was selection of the fittest patients for treatment. Although we corrected for the known confounders functional status and comorbidity, confounding by indication could have resulted in a shorter OS of patients not receiving adjuvant chemotherapy because of unmeasured confounding factors that contraindicated chemotherapy.

The strength of this study was a broad data collection that included specific information about patients, diseases, and treatment-related characteristics. Also, our study included unselected patients, which reflects daily clinical practice. Although not all data were available for all patients, this is the first study to describe the association between diagnosis/treatment of colon cancer and the course of diabetes.

In conclusion, diabetic patients did not have more side-effects due to adjuvant chemotherapy, adjuvant chemotherapy had a positive effect on survival for both patients with and without diabetes, and diabetes control remained good after treatment for cancer. Therefore, diabetes alone is not a reason for withholding adjuvant chemotherapy. Further prospective research should confirm these results.

## Figures and Tables

**Table 1:** Baseline characteristics according to diabetes status.

Characteristic	Frequency (%)		*p* value
	Diabetes (*n*=201)	No diabetes (*n*=206)	
Age (years)			0.12
50–64	39 (19)	57 (28)	
65–69	29 (14)	30 (15)	
70–74	44 (22)	47 (23)	
75–84	77 (38)	56 (27)	
≥85	12 (6)	16 (8)	
Gender			0.38
Male	85 (42)	96 (47)	
Female	116 (58)	110 (53)	
BMI (kg/m^2^)			0.02
<18	1 (<1)	1 (<1)	
18–25	67 (33)	94 (46)	
26–30	55 (27)	51 (25)	
≥31	34 (17)	15 (7)	
Unknown	44 (22)	45 (22)	
ECOG performance status score			<0.01^*^
0+1	118 (62)	147 (75)	
≥2	71 (38)	50 (25)	
Smoking status			0.47
Yes	19 (9)	19 (9)	
No	60 (30)	55 (27)	
Stopped	79 (39)	74 (36)	
Unknown	43 (21)	58 (28)	
Comorbid conditions (*n*) (DM not included)			<0.01
0	39 (19)	80 (39)	
1	65 (32)	68 (33)	
2	58 (29)	39 (19)	
≥3	39 (19)	19 (9)	
Cardiovascular disease			<0.01
Yes	89 (44)	60 (29)	
No	112 (56)	146 (71)	

*p value was determined without missing data. BMI, body mass index; DM, diabetes mellitus; ECOG, European Cooperative Oncology Group.

**Table 2:** Reason given for withholding adjuvant chemotherapy according to diabetes status.

Reason	Frequency (%)	
	Diabetes (*n*=116)	No diabetes (*n*=82)
Advanced age	25 (22)	24 (29)
Overall performance status	12 (10)	9 (11)
Patient’s choice	20 (17)	12 (15)
Comorbidity	17 (15)	2 (2)
Patient died before receiving chemotherapy	13 (11)	8 (10)
Other	3 (4)	0 (0)
Unknown	26 (22)	27 (33)

**Table 3:** Recurrence rate of colon cancer in patients with and without adjuvant chemotherapy treatment according to diabetes status.*

	Rate (%)		*p* value
	Diabetes	No diabetes	
Chemotherapy	28/82 (34)	51/121 (42)	0.25
No chemotherapy	47/108 (44)	38/76 (50)	0.39

*Recurrence status was unknown in 11 diabetic patients and in nine non-diabetic patients. Recurrence percentage was calculated as the number of patients with recurrence divided by the total number of patients at risk of recurrence in the respective group.

**Table 4:** Hazard ratio for all-cause mortality in all patients with colon cancer following univariate and multivariate analyses.

Variable	HR (95% CI)	
	Univariate analysis	Multivariate analysis
Diabetes vs. no diabetes	1.31 (0.99–1.72)	0.99 (0.74–1.32)
Age at time of colon cancer diagnosis	1.05 (1.04–1.07)	1.02 (1.0–1.04)
ECOG score ≥2 vs. 0+1	1.99 (1.50–2.65)	1.37 (0.99–1.89)
Chemotherapy vs. no chemotherapy	0.37 (0.28–0.49)	0.52 (0.35–0.76)
Male vs. female	0.85 (0.64–1.13)	1.03 (0.77–1.39)
Comorbidity frequency		
1 vs. 0	1.28 (0.90–1.83)	1.20 (0.83–1.75)
2 vs. 0	1.15 (0.77–1.72)	0.87 (0.86–1.33)
≥3 vs. 0	2.07 (1.36–3.17)	1.31 (0.83–2.07)

CI, confidence interval; ECOG, European Cooperative Oncology Group; HR, hazard ratio.

**Table 5:** Hazard ratio for all-cause mortality in diabetic patients with colon cancer.

Variable	Univariate analysis	
	HR	95% CI
Duration of diabetes 1–10 years vs. >10 years	0.86	0.54–1.38
Mean HbA1c (%)		
<6.5 vs. 6.5–7.5	1.03	0.57–1.86
7.6–9.0 vs. 6.5–7.5	1.11	0.65–1.88
≥9.1 vs. 6.5–7.5	0.79	0.35–1.76
Use of insulin vs. no insulin	0.91	0.58–1.41
Use of metformin vs. no metformin	0.73	0.49–1.11

CI, confidence interval; HR, hazard ratio.

**Table 6:** Medication for diabetes before and 1 year after colon cancer diagnosis.

Medication	Frequency (%)	
	Before diagnosis	After diagnosis
Diet	9 (5)	9 (5)
Oral antiglycaemic agent	135 (67)	97 (48)
Insulin	43 (21)	44 (22)
Insulin and oral antiglycaemic agent	9 (5)	10 (5)
Unknown or not applicable	3 (2)	41 (20)
